# NHC Polymeric Particles Obtained by Self-Assembly and Click Approach of Calix[4]Arene Amphiphiles as Support for Catalytically Active Pd Nanoclusters

**DOI:** 10.3390/molecules26226864

**Published:** 2021-11-14

**Authors:** Vladimir Burilov, Diana Mironova, Elsa Sultanova, Ramila Garipova, Vladimir Evtugyn, Svetlana Solovieva, Igor Antipin

**Affiliations:** 1 Alexander Butlerov Institute of Chemistry, Kazan Federal University, 18 Kremlevskaya Str., 420008 Kazan, Russia; mir_din@mail.ru (D.M.); elsultanova123@gmail.com (E.S.); aukhadieva.ramilya@yandex.ru (R.G.); vevtugyn@gmail.com (V.E.); iantipin54@yandex.ru (I.A.); 2 A.E.Arbuzov Institute of Organic & Physical Chemistry, 8 Arbuzov Str., 420008 Kazan, Russia; evgersol@yandex.ru

**Keywords:** calixarene, CuAAC, polyNHC, Pd nanoclusters

## Abstract

A new polymeric NHC carrier was synthesized by sequential supramolecular self-assembly and copper-catalyzed azide-alkyne cycloaddition (CuAAC) of amphiphilic imidazolium calix[4]arenes with octyl lipophilic fragments. Obtained polytriazole-imidazolium particles were found as monodisperse submicron particles, with the average diameter of 236 ± 34 nm and average molecular weight of 1380 ± 96 kDa. Successful CuAAC polymerization has been proved using IR spectroscopy and high-resolution ESI mass spectrometry. Polymeric particles, as well as aggregates made from precursor macrocycles, were decorated by Pd clusters (2 nm) for further catalytic investigations. Pd nanoclusters, supported on the polymeric surface, were found highly catalytically active in the model reduction of *p*-nitrophenol, giving reaction rates an order of magnitude higher compared to literature examples. The reaction was recycled using the same catalyst five times without any loss of activity.

## 1. Introduction

During the last few decades, N-heterocyclic carbene (NHC) ligands have attracted considerable attention, owing to their high stability and outstanding applications in homogeneous catalysis [1,2,3]. The advantage of this class of ligands is the ease of their generation through deprotonation of appropriate precursors (most often, imidazolium salts are used) and easily regulated steric/electron donor properties of the ligand by substituents on the heterocyclic precursor core [4,5,6,7]. Amongst N-heterocyclic carbenes, polytopic ligands featuring more than one NHC unit are of high demand, as they impact the stability of the final metal complex, owing to their chelating abilities [8,9]. A special case of polytopic NHC ligands are polymeric NHCs [10], which can be obtained by grafting on a polymeric support [11] or by copolymerization of imidazolium—containing monomer to give self-supported polymeric NHC [12,13,14]. Such polymeric NHCs combine the activity and selectivity of homogeneous catalysts, with the possibility of reuse, which is characteristic for heterogeneous ones.

**Scheme 1:**
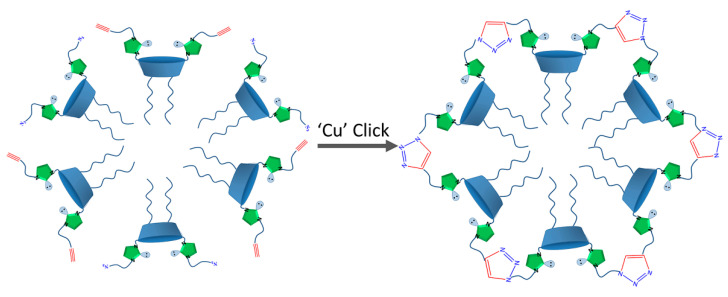
Representation of the formation of polymeric nanoparticles using a supramolecular approach.

Recently [15], we proposed a new approach to the formation of polymeric NHC carriers based on sequential supramolecular self-assembly of amphiphilic calix[4]arenes containing azidoalkyl/alkynyl fragments on the polar region of macrocycles into aggregates in an aqueous solution, followed by foregoing cross-linking of macrocycles using a copper-catalyzed azide-alkyne cycloaddition (CuAAC) reaction (Scheme 1).

However, due to the rather low lipophilicity of the macrocycles, insufficient for the formation of a stable core during polymerization, in addition to spherical nanoparticles, the formation of open-chain polymers was also found. Calixarenes, an established and well-known class of macrocycles [16,17,18], are very promising building blocks for creating such polymer systems. Hydrophobic and hydrophilic fragments, including those containing anchor groups for polymerization, can be easily introduced into their structure. The presence of imidazolium fragments, fixed on the macrocyclic platform, makes it possible to obtain catalytically active amphiphilic bis-NHC Pd(II) complexes, which we have shown earlier [19,20].

Moving forward a supramolecular approach in the creation of structured polymer particles, in the presented article, we propose the synthesis of more lipophilic azide and alkyne calix[4]arene derivatives to exclude the possibility of the formation of open-chain polymers, as well as the preparation of structured polymer particles based on them.

## 2. Results and Discussion

**Scheme 2:**
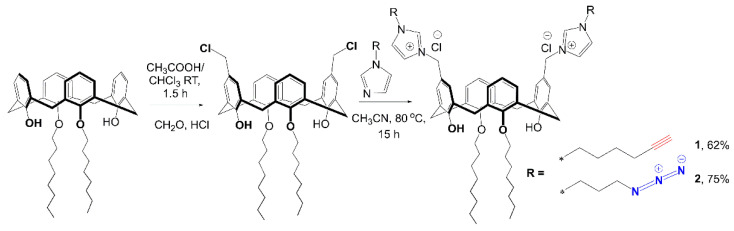
Synthetic pathway for macrocycles **1** and **2** containing azidoalkylimidazolium/alkynylimidazolium groups on the upper rim.

First, an alkyne-terminated calixarene **1** and bis-azide calixarene **2** were synthesized according to our previously reported approach [21]. For this, starting *p*-*H*-calix[4]arene was obtained by de-*tert*-butylation of *p-tert*-butylcalix[4]arene in toluene in the presence of AlCl_3_ and phenol [22]. Then, *p-H*-calix[4]arene was alkylated with 1-bromoctane according to the Williamson reaction to give di-O-octyl derivative, which was involved in the Blanc reaction to derive bis-chloromethylated calix[4]arene (Scheme 2) [23].

For the synthesis of the final compounds, bis-chloromethylated calix[4]arene and 1-(hex-5-yn-1-yl)-1H-imidazole or 1-(3-azidopropyl)-1H-imidazole were dissolved in acetonitrile and were boiled until the bis-chloromethylated calixarene disappeared. The described strategy allows the introduction of any alkyl/imidazolium fragments into the lower/upper rims of the calix[4]arene macrocyclic platform with high yields. All spectral data for **2** matched previously published data [21]. The structure of **1** was proven using NMR ^1^H, ^13^C, IR, and high-resolution ESI (HRESI) mass spectrometry (Supplementary Materials Figure S1). It is noteworthy that, even with the use of such a mild ionizing method as electrospray, in HRESI spectra, in addition to the peak of the molecular ion corresponding to the (M-2Cl)^2+^ (*m*/*z* calculated for C_64_H_82_N_4_O_4_^2+^ = 485.3163, found 485.3177), the presence of various variations of quinone methide structures, as well as structures with a loss of hexynyl fragment, was found (the corresponding fragment structures are given in Supplementary Materials Table S1). The data obtained agree with those previously obtained by the MALDI method: similar structures easily cleave off imidazolium fragments, converting to the corresponding quinone methides [15].

### 2.1. Aggregation and CuAAC Polymerization of the Core

The next step was the study of aggregation abilities of **1**, **2** and their mixtures in aqueous solutions. A pyrene probe was used to determine the critical aggregation concentration (CAC) values of macrocycles **1**, **2** (Table 1, Supplementary Materials Figure S2). Interestingly, macrocycle **2** has a quite significant CAC value compared to **1**. The same observation was found in the case of less lipophilic butyl calixarene derivatives earlier [15]. High CAC value may be a result of an increase in the volume of the headgroup of the macrocycle thanks to the spreading alkylazide groups. In a mixture of **1** and **2**, the CAC value averages between them.

The polymerization of calixarenes **1** and **2** was performed using a two-step approach combining self-assembly and a surface cross-linking step. First, 0.1 mM solutions of macrocycles **1** and **2** were self-assembled in water under stirring. After assembly, the obtained aggregates were covalently cross-linked to hold the micelle-like structure intact. Cross-linking was performed using 25 mol% of CuCl_2_ and 50 mol% of sodium ascorbate in deionized water during 12 h at 25 °C. The resulting particles were purified by dialysis against a solution of Trilon B (0.01 M), and then against deionized water. According to the dynamic light scattering (DLS) data (Table 1), after cross-linking, the size of the particles slightly increased, whereas their polydispersity index remained unchanged.

**Figure 1:**
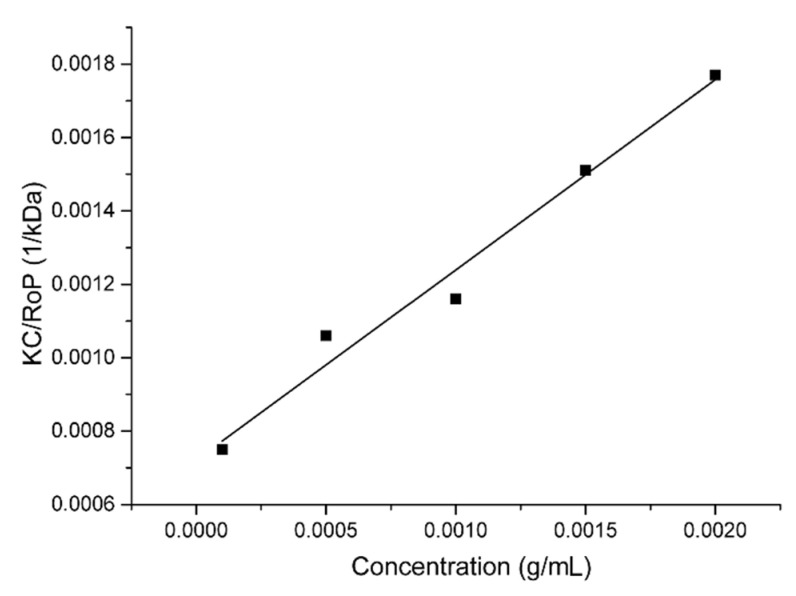
Debye plot of **1** + **2** mixture after CuAAC polymerization (0.01–2 mg/mL).

According to the static light scattering (SLS) data (Figure 1), the cross-linked particles of **1** and **2** have an average molecular weight of 1380 ± 96 kDa. Taking into account both DLS and SLS data, there can be assumed a lack of a side formation of open-chain polymers.

**Figure 2:**
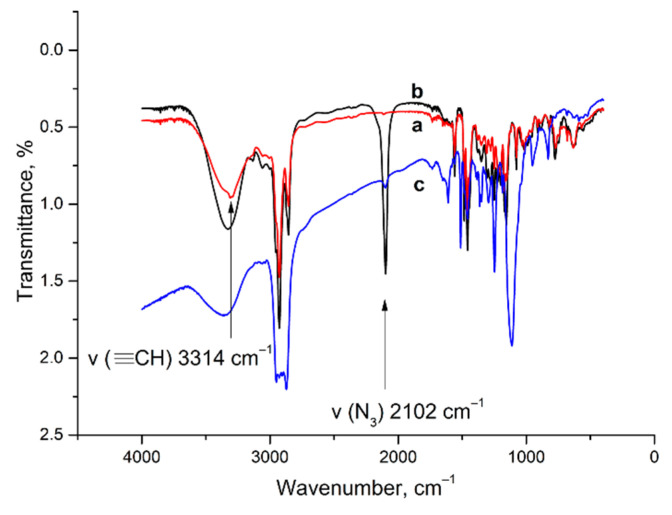
IR spectra (KBr pellets) of **1**(**a**), **2**(**b**), and mixture of **1**, **2** after cross-linkage (**c**).

**Figure 3:**
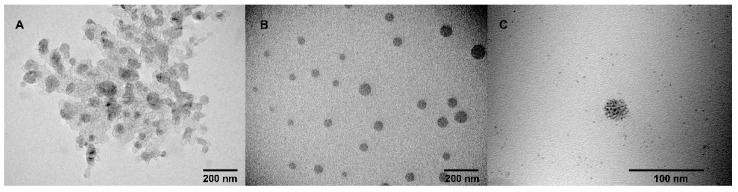
TEM images of empty polymeric particles (**1** + **2**) (**A**) and polymeric particles decorated with Pd (**B**,**C**).

The resulting particles were then dried and characterized by IR spectrometry. An intense band at 2102 cm^−1^, related to the stretching vibrations of the azide group in the spectrum of the initial azide **2** (Figure 2 curve b), completely disappeared in the spectra of cross-linked polymer (Figure 2 curve c). The same changes were found when comparing to the initial IR spectra of alkyne **1**: the shoulder at 3314 cm^−1^, related to the terminal alkynyl C–H bond stretching vibrations (Figure 2 curve a), also disappeared in the polymer spectra. The results of transmission electron microscopy (TEM) study indicate that polymeric particles have a spherical shape, with an average diameter around 50–70 nm (Figure 3A).

**Figure 4:**
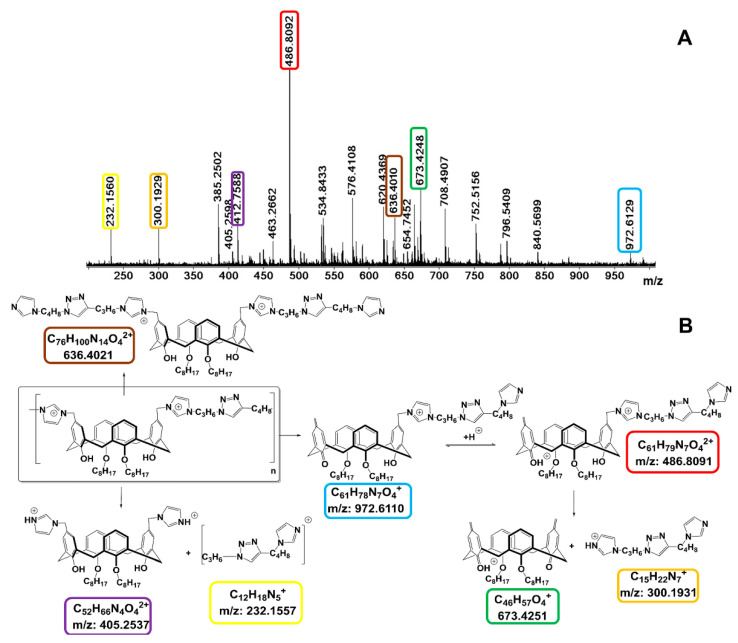
HRESI MS spectra in MS mode of aqueous dispersion of polymeric particles made by CuAAC reaction of **1** and **2** (**A**), and corresponding fragmentation patterns of polymer (**B**).

Polymeric particles were studied using HRESI MS techniques. Electrospray ionization is a soft ionization technique and is widely used to ionize different organic analytes, including thermally labile ones [24]. Despite the fact that ions generated through ESI ionization possess low internal energy and usually fragmentation in a single-stage MS experiment is negligible [25], the recorded spectrum shows a significant fragmentation process (Figure 4A). The main direction of the fragmentation of polymer is formation of mono-cation of quinone methide adduct with one imidazolium-triazole spacer with *m*/*z* = 972.6129 (calculated for C_61_H_78_N_7_O_4_^+^ = 972.6110), as well as di-cation with *m*/*z* = 486.8092 (calculated for C_61_H_79_N_7_O_4_^2+^ = 486.8091) (Figure 4B). Quinone methide adduct with m/z = 972.6129 undergoes subsequent fragmentation, with formation of bis-quinone-methide with *m*/*z* = 673.4248 (calculated for C_46_H_57_O_4_^+^ = 673.4251) and imidazolium-triazole spacer with *m*/*z* = 300.1929 (calculated for C_15_H_22_N_7_^+^ = 300.1931). Moreover, a low intensive but also very important peak corresponding to one calixarene with two imidazolium-triazole spacers with *m*/*z* = 636.4010 (calculated for C_76_H_100_N_14_O_4_^2+^ = 636.4021) was found in spectra. Besides the quinone methide route, two products of cleavage of the C–N bond of imidazolium and propyltriazole with *m*/*z* = 405.2598 (calculated for C_52_H_66_N_4_O_4_^2+^ = 405.2537) and *m*/*z* = 232.1560 (calculated for C_12_H_18_N_5_^+^ = 232.1557) were found. Such dequaternization reactions are usually dominated in ESI ionization-initiated fragmentation of quaternary compounds [26]. Thus, the presented fragmentation scheme fully proves the successful course of CuAAC polymerization with the formation of triazole linkers in the structure of the polymer.

### 2.2. Decoration of the Core by Pd and Catalytic Activities of Received Nanoparticles in Model Reduction Reaction of p-Nitrophenol

Obtained polymeric particles were decorated by Pd for further catalytic investigations. Ascorbic acid was used as a well-known green reductant, usually used for the synthesis of small Pd nanoclusters [27]. For this, under vigorous stirring, Na_2_PdCl_4_ was mixed with aqueous dispersion of polymeric particles (1 mM of Na_2_PdCl_4_ and 0.05 mM of particles), and then Pd(II) was reduced by 10 equivalents of ascorbic acid under vigorous stirring for 2 h. The obtained solution turned black, confirming the formation of metallic palladium. According to the TEM data (Figure 3B,C), palladate ions are reduced to palladium nanoclusters of 2 nm, which are organized on the polymeric surface. The presence of palladium nanoparticles on the surface of polymeric particles has been proven by energy dispersive X-ray analysis (EDX) (Supplementary Materials Figure S3). At EDX spectra, there are signals of C, N, and O of the organic core, as well as signals of Pd on the surface. To compare the difference between polymeric support with nonpolymeric support, macrocycles **1**, **2** and their mixture, as well as unsupported Pd0 nanoparticles, were synthesized. All supported Pd nanoparticles were found to be colloidally stable upon staying for 1 week (Supplementary Materials Figure S4), whereas unsupported Pd0 fully sedimentated. According to the DLS and ELS data (Table 2), unsupported Pd0 have a very high polydispersity index with low ζ potential, while all other systems have much more colloidal stability. Interestingly, decoration of all studied systems with Pd0 led to the compaction of the aggregates. One of the reasons for this can be partial formation of noncharged carbene species from imidazolium units in polymer and a subsequent decrease in repulsion between neighboring polymeric links. This is indicated by a decrease in the surface potential of aggregates (Table 2).

**Figure 5:**
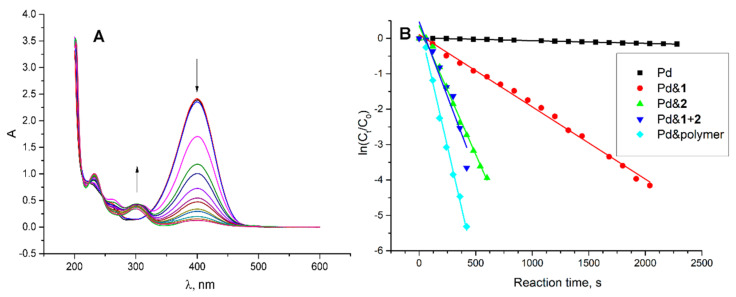
Time-dependent UV/Vis absorption spectra of test reaction mixture in the presence of Pd and polymer (**A**); plots of ln(C_t_/C_0_) vs. catalytic reaction time for different Pd-containing catalytic systems (**B**). C(PNP) = 0.1mM, C(NaBH_4_) = 5 mM, C(**1, 2**, polymer, Pd) = 0.002 mM, H_2_O, 25 °C.

The comparative catalytic activities of palladium-decorated polymeric particles were investigated using a model reduction reaction of *p*-nitrophenol (PNP) into *p*-aminophenol (PAP) in the presence of NaBH_4_. The reduction reaction can be easily monitored by UV/Vis spectroscopy by monitoring the intensities of the absorption bands at 400 (absorbance of PNP) and 300 nm (absorbance of PAP) [28]. The characteristic peak of PNP at 400 nm, which is ascribed to the formation of the *p*-nitrophenolate ion, gradually decreased, while a new peak at 300 nm, corresponding to PAP, started to appear (Figure 5). In the absence of a catalytic system, PNP to PAP transformation was not initiated. Figure 5B shows plots of ln (C_t_/C_0_) versus time for the reduction of PNP, where C_0_ and C_t_ are the initial concentration of PNP and its concentration at time t, respectively.

The calculated rate constants and specific catalytic activity were estimated from diffusion-coupled first-order reaction kinetics using the slopes of straight lines in Figure 5B, and are presented in Table 3.

Unsupported Pd nanoparticles turned out to be inactive in comparison with the stabilized systems. Stabilization of Pd nanoparticles, even on polymerized macrocycles, led to an increase in SAA by one or even two orders of magnitude in the case of Pd and **2**, and Pd and **1** + **2.** The use of polymeric support was found to be the most successful and had given the maximal SSA value. There are several reasons for the high catalytic activity of polymer-supported Pd nanoclusters. Firstly, polymer particles are more rigid compared to non-polymerized ones. Therefore, the rearrangement of the catalyst, as well as re-coordination of palladium particles giving palladium aggregates during the reaction, is minimized. The second reason is the formation of triazole fragments in addition to the NHC fragments in the polymer. The proximity of triazole and NHC ligands leads to a chelating effect [29], thus increasing the efficiency of the ligand–metal interactions and preventing the aggregation of palladium particles into inactive agglomerates. Taking into account very low Pd loading (3.2 nanomoles), the obtained catalytic systems outperform recently published systems. For example, Pd–protein nanocomposites, obtained by Yu et al. [30], demonstrate RC in the PNP reduction as 0.005 s^−1^, which is an order of magnitude lower than that of Pd and polymer system (0.0132 s^−1^). Pd, supported on chitosan–Fe_3_O_4_ nanocomposite obtained by Veisi et al. [31], also demonstrates lower rates in the PNP reduction (0.0026 s^−1^).

**Figure 6:**
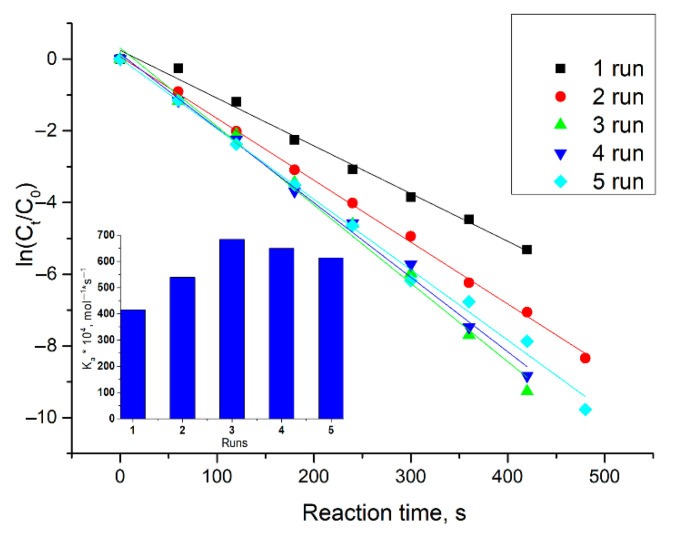
Plots of ln (C_t_/C_0_) vs. catalytic reaction time for Pd and polymer catalytic reduction of PNP (5 cycles). C(PNP) = 0.1mM, C(NaBH_4_) = 5 mM, C(polymer, Pd, = 0.002 mM, H_2_O, 25 °C). Insert: K_a_ values for different catalytic cycles.

High recyclability and durability of catalysts is a critical factor that should be evaluated in developing catalysts for further industrial usage. To test the recyclability of the Pd and polymer catalytic system, we carried out multicyclic reactions by repeatedly using the same Pd and polymer catalyst. Considering the small size of the system, filtration or centrifugation would lead to significant losses of the Pd and polymer. In order to eliminate the loss of catalyst, it was decided to carry out a model reduction reaction in one cell. For this, a new portion of PNP was added after the full conversion of the previous one with the 10 min time gap. According to the obtained data (Figure 6, Table 3), in the second and third cycles, the activity of the catalyst even increased, and then remained unchanged. The catalytic reduction of PNP takes place on the surface of the palladium nanoparticles, and all reactants (PNP and borohydride ion) must be adsorbed on the surface of nanoparticles to react [32]. It is known that positively charged support of metal nanoparticles facilitates absorption of both borohydride anion, as well as PNP anion, thus increasing reaction rates [33]. Thus, the increase in catalytic activity starting from the second cycle is most likely associated with the adsorption of the *p*-aminophenol (which is positively charged in a neutral and acidic pH) on the catalyst surface.

## 3. Methods

### 3.1. Dynamic, Static, and Electrophoretic Light Scattering (DLS, SLS, and ELS)

DLS, SLS, and ELS experiments were carried out on a Zetasizer Nano ZS instrument (Malvern Panalytical, Worcestershire, UK) with 4 mW 633 nm He–Ne laser light source and the light scattering angle of 173°. The data were treated with DTS software (Dispersion Technology Software 5.00). The solutions were filtered through a 0.8 μM filter before the measurements to remove dust. The experiments were carried out in disposable plastic cells DTS 0012 (size), standard glass cells (10 mm, molecular weight), or in disposable folded capillary cells DTS 1070 (zeta potential) (Sigma–Aldrich, St. Louis, MI, USA) at 298K, with at least three experiments for each system. Statistical data treatment was carried out using t-Student coefficient and the particle size determination error was <2%. The prepared samples were ultrasonicated within 30 min at 25 °C before measurements.

### 3.2. Critical Aggregation Concentration (CAC) Determination

CAC values were measured using a pyrene fluorescent probe and calculated from the dependence of the intensity ratio of the first (373 nm) and third (384 nm) bands in the emission spectrum of pyrene vs. calixarene concentration. Fluorescence experiments with pyrene were performed in 10.0 mm quartz cuvettes and recorded on a Fluorolog FL-221 spectrofluorometer (Horiba, Ltd., Kyoto, Japan) in the range of 350 to 430 nm and excitation wavelength of 335 nm with a 2.5 nm slit. All studies were conducted in aqueous solution at 298 K.

### 3.3. Transition Electron Microscopy (TEM)

TEM was performed on a Hitachi HT7700 ExaLens (Hitachi High-Tech Corporation, Tokyo, Japan) in Interdisciplinary Center for Analytical Microscopy of Kazan Federal University. The images were acquired at an accelerating voltage of 100 kV. Samples were ultrasonicated in water for 10 min, dispersed on 200 mesh copper grids with continuous formvar support films, and then dried during 3 h. Energy dispersive X-ray spectroscopy was performed using an Oxford Instruments X-MaxN 80T detector (Oxford Instruments, Abingdon, UK).

### 3.4. IR Spectra

The IR spectra were recorded on a Bruker Vector-22 spectrometer (Bruker Corporation, Bill Rica, MA, USA). Powdered samples were mixed with KBr and then pressed in a press form to give a transparent KBr tablet.

### 3.5. HRESI Mass Spectrometry

HRESI experiments were performed at Agilent 6550 iFunnel Q-TOF LC/MS (Agilent Technologies, Santa Clara, CA, USA) in the positive mode, using internal calibration for better accuracy of masses.

### 3.6. Refractometry

The dependence of the refractive index of the polymer on the concentration (dn/dc) required for determining the molecular weight by the method of static light scattering was determined on an automatic digital refractometer Atago RX-7000i (Atago Co., Ltd., Tokyo, Japan) at 25 °C.

### 3.7. Catalytic Reduction of PNP

The solution had been purged prior to the run with Ar gas in order to remove O_2_. In a typical run, to a suspension of palladium-decorated polymeric particles (2 µM) dispersed in H_2_O, a freshly prepared aqueous solution of NaBH_4_ (5000 µM) was added and the mixture was stirred for 15 min at an adjusted temperature (298 K). Then, PNP (100 µM) was added to the mixture and the suspended solution was carefully mixed by shaking gently before measurements. The reaction progress was monitored by assessing a small portion after filtration of the reaction mixture at regular time intervals and measuring the optical density of the reaction mixture, after diluting 10 times, at 400 nm as a function of time. Measurements were carried out using a Shimadzu UV-2600 spectrophotometer (Shimadzu Corporation, Kyoto, Japan) in quartz cell (light path 10 mm).

## 4. Materials

TLC was performed on Merck UV 254 plates with a Vilber Lourmat VL-6.LC UV lamp (254 nm) control (Vilber, Marne-la-Vallée, France). NMR spectra were recorded on a Bruker Avance 400 Nanobay (Bruker Corporation, MA, USA) with signals from residual protons of DMSO-d6 as internal standard. The melting points were measured using the OIptimelt MPA100 melting point apparatus (Stanford Research Systems, Sunnyvale, CA, USA). All reagents were purchased from either Acros or Sigma-Aldrich and used without further purification. Solvents were purified according to standard methods [34]. Hex-5-yn-1-yl 4-methylbenzenesulfonate [35], 1-(hex 5-yn-1-yl)-1H-imidazole [15], 11,23-Bis(chloromethyl)-25,27-dihydroxy-26,28-dibutoxycalix[4]arene [21], and 11,23-Bis[3(1-(3-azidopropyl))-1H-imidazolium]-25,27-dihydroxy-26,28-dibutoxycalix[4]arene dichloride **2** [21] were synthesized by previously reported methods.

### 4.1. Synthesis of 11,23-bis[3-(1-(hex-5-yn-1-yl)-1H-imidazolium)methyl]-25,27-dihydroxy-26,28-dioctyloxycalix[4]arene dichloride **1**

In total, 0.16 g (0.21 mmol) of 11,23-bis(chloromethyl)-25,27-dihydroxy-26,28-dioctyloxycalix[4]arene and 0.085 g (0.58 mmol) of 1-(hex-5-yn-1- yl)-1H-imidazole were dissolved in 3.5 mL of dry acetonitrile. Reaction mixture was stirred at 80 °C for 15 h, then solvent was removed on a rotary evaporator and the residue was added to diethyl ether (25 mL). A beige solid was collected by filtration on a sintered glass funnel (0,14 g, 62%).

Melting point: 123 °C (decomp). ^1^H NMR (400 MHz, DMSO-d6, 25 °C) δH ppm: 0.85 br t (6H, CH_3_), 1.26–1.50 m (20H, CH_2_), 1.66–1.77 m (4H, CH_2_), 1.92–1.83 m (4H, CH_2_), 1.94–2.04 m (4H, CH_2_), 2.17–2.26 br m (4H, HC≡C–CH_2_), 2.85 br t (2H, HC≡C), 3.42 d (4H, Ar-CH-Ar), 3.96 br t (4H, CH_2_O), 4.10–4.24 m (8H, CH_2_ + Ar-CH_2_-Ar), 5.21 s (4H, Ar-CH_2_-Im), 6.79 t (2H, HAr, J = 7.3 Hz), 7.05 d (4H, HAr, J = 7.4 Hz), 7.29 s (4H, HAr), 7.80 br s (4H, HIm), 8.67 s (2H, OH), 9.36 s (2H, NH-Im). ^13^C{1H} NMR (100.6 MHz, DMSO-d6, 25 °C) δC ppm: 13.97, 17.20, 22.17, 24.60, 25.44, 28.62, 28.84, 28.94, 29.60, 30.36, 31.42, 48.38, 51.72, 71.80, 76.57, 83.90, 122.47, 122.66, 125.17, 125.48, 128.23, 128.97, 133.46, 135.84, 151.80, 153.17. IR (KBr) ν max cm^−1^: 1459 (Car=CAr), 1485 (CAr-CAr), 2857 νs(–CH_2_–), 2928 ν as(–CH_2_–), 2959 ν as(CH_3_), 3314 (≡C–H).

HRMS (ESI) *m*/*z* [M-2Cl]^2+^ calculated for [C_64_H_82_N_4_O_4_]^2+^ 485.3163, found: 485.3177.

### 4.2. Cross-Linking of 11,23-Bis[3(1-(hex-5-yn-1-yl)-1H-imidazolium]-25,27-dihydroxy-26,28-dioctyloxycalix[4]arene dichloride 1, 11,23-Bis[3(1-(3-azidopropyl))-1H-imidazolium]-25,27-dihydroxy-26,28-dioctyloxycalix[4]arene dichloride 2

In total, 5 mg (100 μmol) of **1** and 5 mg (100 μmol) of **2** were dissolved under vigorous stirring in 50 mL of deionized water at 25 °C. After 10 min, 0.17 mg (25 μmol) of CuCl_2_ and 0.49 mg (50 μmol) of sodium ascorbate were added and the mixture was stirred during 12 h at 25°. After 12 h, the obtained solution was purified using a dialysis membrane (1 kDa cut-off) against 0.01 M Trilon-B (3 × 50 mL) for 8 h, and then against deionized water (3 × 50 L) for 5 h. Water was removed by a rotary evaporator to give polytriazole as white fine powder (79%).

### 4.3. Synthesis of Pd Nanoparticles

In total, 15 μL of 4 mM solution of calixarene (**1** or **2**) (in the case of Pd and **1** or Pd and **2**) or 7.5 μL of both of them (in the case of Pd and **1** + **2**) or 300 μL of 0. 2 mM polymer solution were dissolved in 0.5 mL of Milli-Q water. Then, under vigorous stirring, 60 μL of freshly prepared solution of Na_2_PdCl_4_ (2mM) was added. Finally, 120 μL of 1000 mM solution of ascorbic acid was added and the total volume was increased to 1.2 mL. The mixture was stirred for 2 h at room temperature. The control of the reduction of [PdCl_4_]^2−^ was performed by the disappearance of an absorption band at 410 nm in the UV/Vis spectra.

## 5. Conclusions

Stable polyimidazolium particles, with the average diameter of 236 ± 34 nm in aqueous solutions and average molecular weight of 1380 ± 96 kDa, were made by self-assembly, followed by CuAAC cross-linking of amphiphilic imidazolium calix[4]arenes containing “clickable” azide or alkynyl fragments on the polar region of macrocycles. Obtained polymeric particles were decorated by Pd using reduction of aqueous solution of Na_2_PdCl_4_ by ascorbic acid. Pd nanoclusters of 2 nm, supported on the polymeric surface, were found highly catalytically active in the model reduction of *p*-nitrophenol, giving reaction rates an order of magnitude higher compared to literature examples. The reaction was recycled using the same catalyst five times without any loss of activity. The proposed self-assembly click approach opens up completely new perspectives in the creation of nanomaterials with a controlled structure.

## Figures and Tables

**Table 1:** CAC values, DLS, and ELS data of aggregates formed by macrocycles **1**, **2** and their mixture before and after CuAAC polymerization.

System	CAC, µM	d, nm	PDI	ζ. mV
**1**	24	122 ± 5	0.465 ± 0.045	+54 ± 11
**2**	130	98 ± 9	0.432 ± 0.124	+97 ± 1
**1** + **2**	40	167 ± 22	0.424 ± 0.043	+70 ± 10
polymer		236 ± 34	0.401 ± 0.043	+66 ± 10

C (pyrene) in CAC experiment = 1 µM, C (**1** = **2**) = 0.1 mM.

**Table 2:** DLS and ELS data of aqueous solutions of Pd nanoparticles alone and stabilized by macrocycles **1**, **2** and their mixture before and after CuAAC polymerization.

System	d, nm	PDI	ζ. mV
Pd0	230 ± 21	0.967 ± 0.058	+2 ± 0.6
Pd & **1**	61 ± 1	0.322 ± 0.001	+66 ± 2
Pd & **2**	93 ± 17	0.261 ± 0.096	+58 ± 7
Pd & **1** + **2**	65 ± 2	0.438 ± 0.073	+62 ± 9
Pd & polymer	198 ± 7	0.382 ± 0.073	+60 ± 6

**Table 3:** Rates of reaction (RC) and specific catalytic activity (SAA) for the reduction in PNP using different catalytic systems.

Catalytic System	RC, *k*, × 10^2^ s^−1^	SAA, *K_a_*, × 10^4^ mol^−1^s^−1^
Pd0	0.012 ± 0.002	4 ± 0.6
Pd and **1**	0.198 ± 0.016	62 ± 5
Pd and **2**	0.771 ± 0.023	241 ± 7
Pd and **1** + **2**	0.950 ± 0.070	297 ± 22
Pd and polymer 1 run	1.328 ± 0.099	415 ± 31
Pd and polymer 2 run	1.724 ± 0.038	539 ± 12
Pd and polymer 3 run	2.185 ± 0.035	683 ± 11
Pd and polymer 4 run	2.076 ± 0.016	649 ± 5
Pd and polymer 5 run	1.964 ± 0.048	614 ± 15

## Data Availability

Not applicable.

## Supplementary Materials

The following materials are available online: NMR, HRESI MS spectra of **1**; fragmentation table for HRESI spectra of **1;** Pyrene plots for **1**, **2** and **1**+**2**; EDX spectra of Pd nanoparticles and photographs of stabilized Pd nanoparticles solutions.

## Sample Availability

Samples of the compounds **1**, **2**, as well as polymeric particles, including those decorated by Pd, are available from the authors.
