# Supplementary material for: NHC Polymeric Particles Obtained by Self-Assembly and Click Approach of Calix[4]Arene Amphiphiles as Support for Catalytically Active Pd Nanoclusters
Source: Molecules. 2021 Nov 14;26(22):6864. doi: 10.3390/molecules26226864 (PMC8625650; doi:10.3390/molecules26226864)
Supplement: Supplementary file 1 [file molecules-26-06864-s001.zip › molecules-1429107-supplementary.pdf]

NHC polymeric particles obtained by self assembly&click approach of calix[4]arene amphiphiles as support for catalytically active Pd nanoclusters

Vladimir Burilov,[1*] Diana Mironova,[1] Elsa Sultanova,[1] Ramila Garipova,[1] Evtugyn Vladimir,[1] Svetlana Solovieva[2] and Igor Antipin[1]

[1.] Kazan Federal University, 18 Kremlevskaya st. Kazan, 420008, Russian Federation.
[2.] A.E.Arbuzov Institute of Organic & Physical Chemistry, 8 Arbuzov str., Kazan, 420088, Russian Federation.
* Correspondence: ultrav@bk.ru; Tel.: (+7-843-2337344)

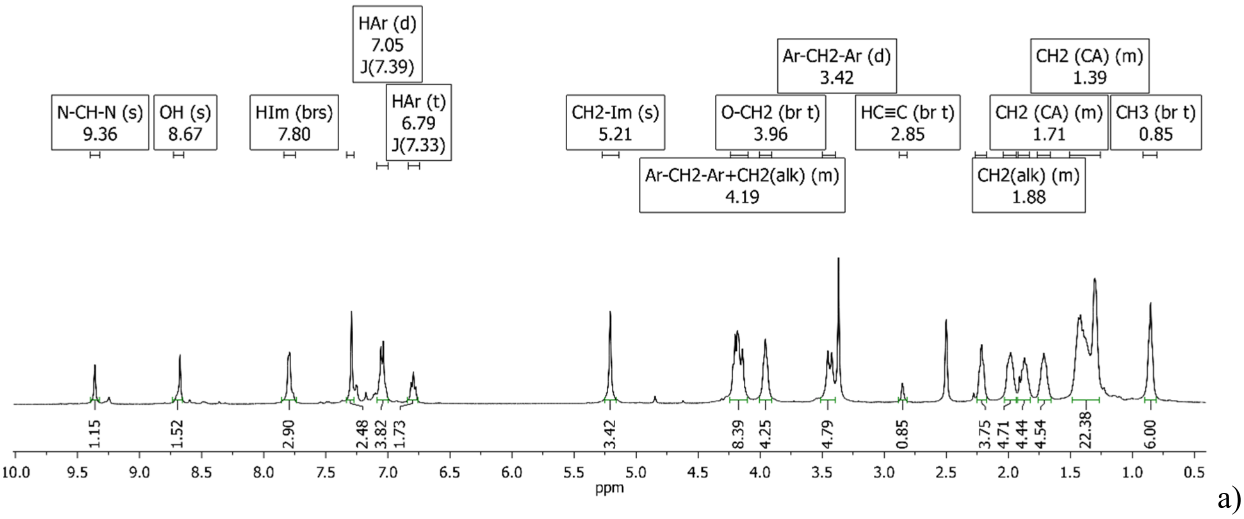

a)

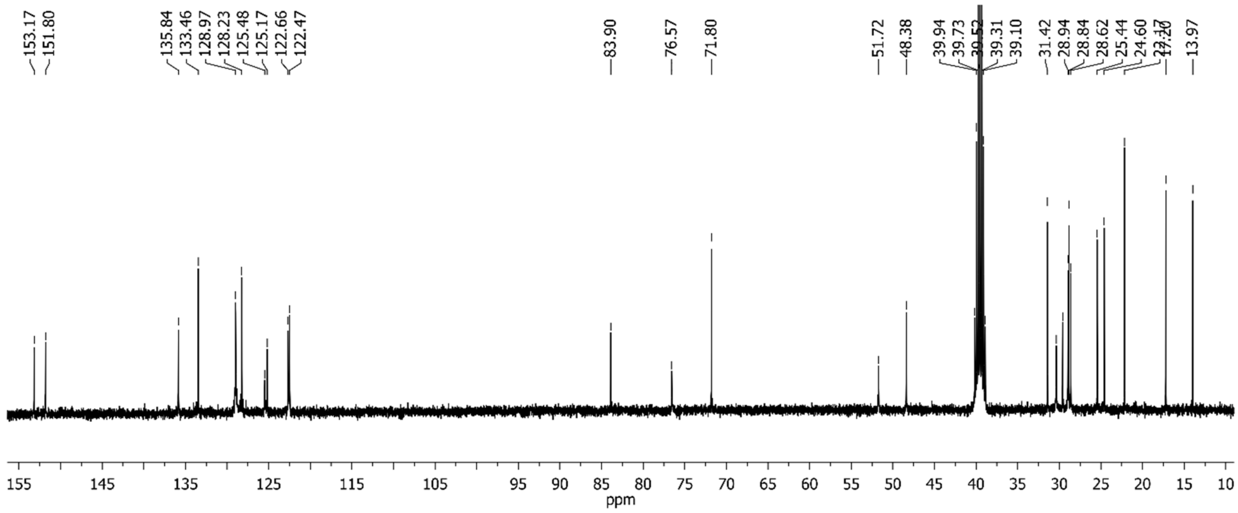

b)

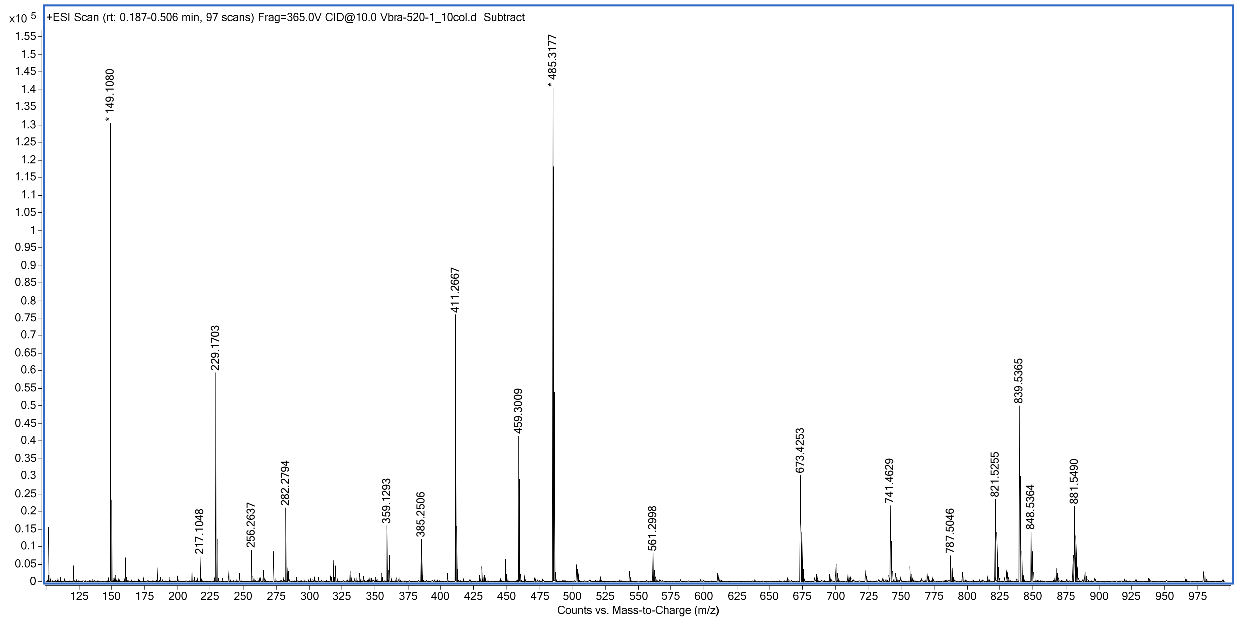

c)

Figure S1. NMR ^{1}H (a), ^{13}C (b) and HRESI MS (c) spectra of 11,23-bis[3-(1-(hex-5-yn-1-yl)-1H-imidazolium)methyl]-25,27- dihy-droxy-26,28-dioctyloxycalix[4]arene dichloride 1.

Table S1. Fragmentation table for HRESI spectra of **1**.

Structure	m/z found	m/z calculated
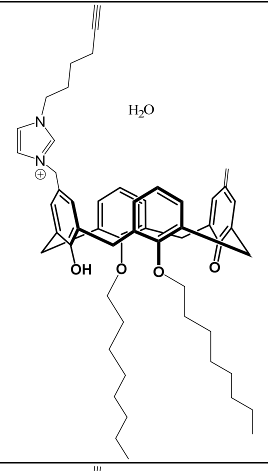	839.5365	839,5357
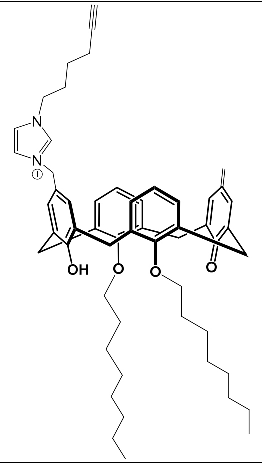	821.5255	821.5252
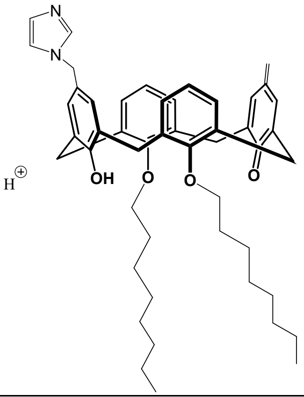	741.4629	741.4626
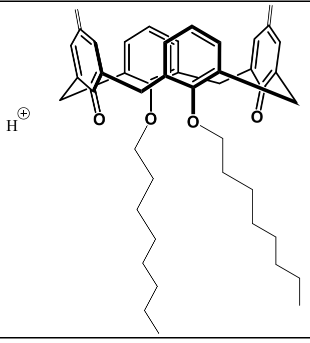	673.4253	673.4251

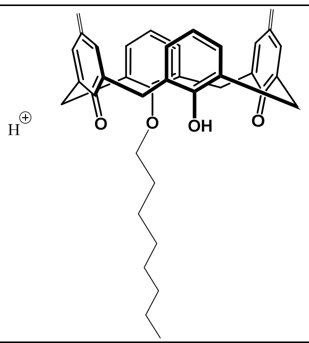	561.2998	561.2999
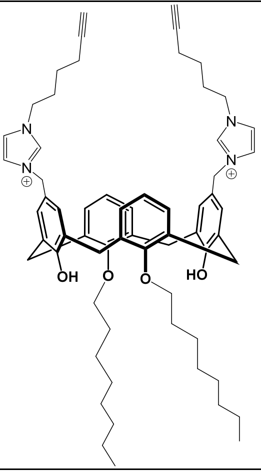	485.3177	485.3163
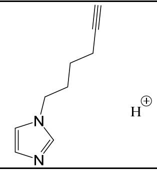	149.1080	149.1073
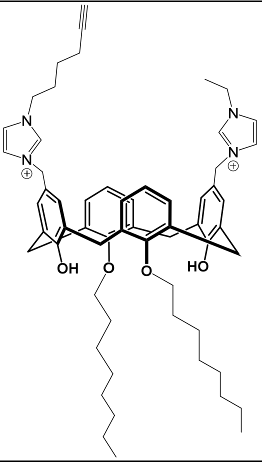	459.3009	459.3006

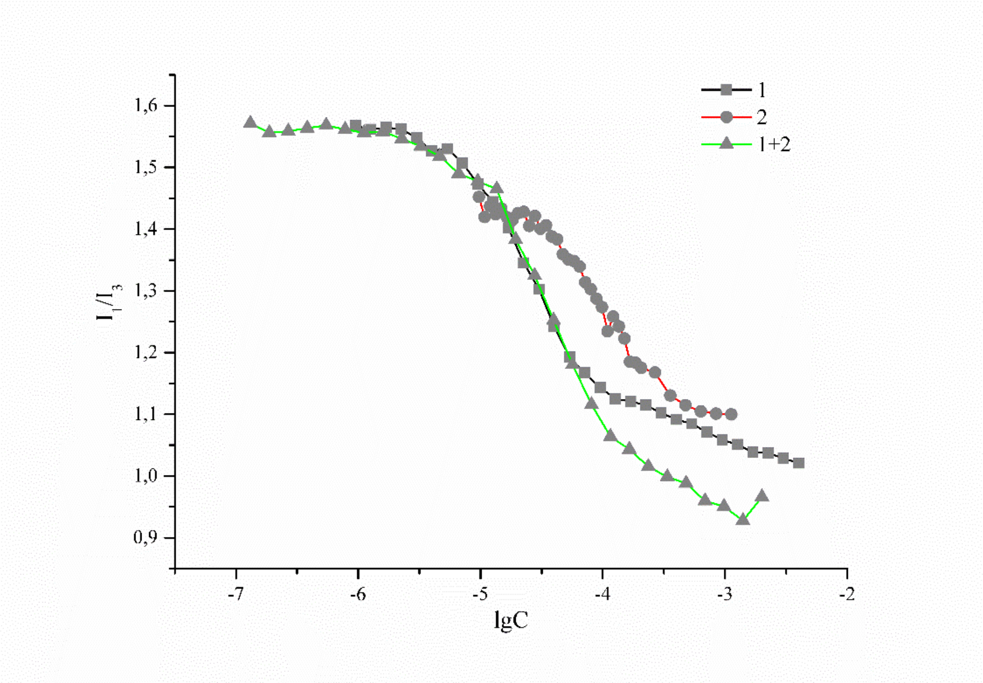

Figure S2. Dependence of I1/I3 pyrene ratio vs. lg(C) of **1**, **2**, and their mixtures, C (pyrene) = 1 μM, ultrapure water, 25 °C.

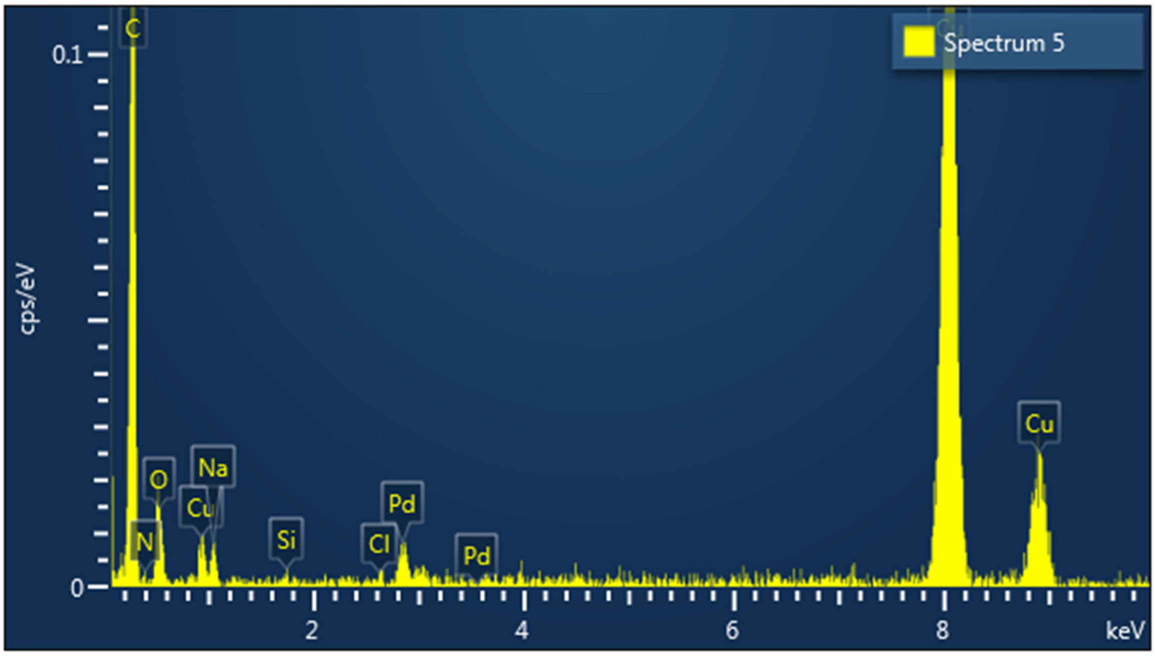

Figure S3. EDX spectra of Pd nanoparticles, stabilized on the surface of **1** + **2** polymer.

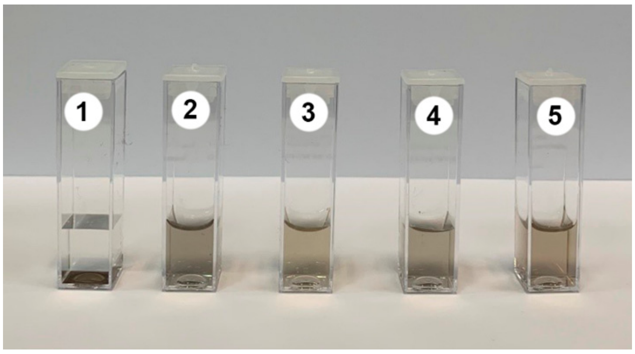

Figure S4. Photography of cuvettes containing Pd (1), Pd&**1** (2), Pd&**2** (3), Pd&**1**+**2** (4) and Pd&polymer (5)
